# Willingness to Stop Growing Tobacco in Uganda

**DOI:** 10.1200/JGO.18.00242

**Published:** 2019-04-03

**Authors:** Adelaine Karemani, Fred Nuwaha

**Affiliations:** ^1^ Makerere University School of Public Health, Kampala, Uganda

## Abstract

**PURPOSE:**

Tobacco use is the leading cause of premature death worldwide. One aspect of tobacco control is convincing farmers to stop tobacco production. We assessed the willingness of tobacco farmers in Uganda to stop growing tobacco.

**METHODS:**

We conducted a cross-sectional interview survey with an interviewer-administered questionnaire. Participants were active tobacco farmers in 12 villages of Kanungu district (N = 528) and were interviewed in 2013 to assess their willingness to stop growing tobacco.

**RESULTS:**

Most farmers (61.7%) grew tobacco only as a cash crop with no supplemental income. A total of 198 farmers (37.5%) were willing to stop growing tobacco. Ninety-two respondents cited coffee as a potential replacement crop for tobacco and 106 mentioned rice. Barriers to growing replacement crops were that tobacco is highly profitable (n = 172) and that the soil (n = 175) and/or weather (n = 22) cannot sustain any crops other than tobacco. Willingness to stop growing tobacco was 1.32 times as likely among farmers who said that tobacco use causes respiratory diseases and 1.16 times as likely among farmers who received less than less than $300 USD from tobacco sales in the previous season. Agreeing that tobacco is profitable decreased the likelihood of willingness to stop growing tobacco by 36%.

**CONCLUSION:**

Only one in three farmers were willing to stop growing tobacco. To increase their willingness to stop growing tobacco, farmers must be educated on the health consequences of tobacco and we must demonstrate to farmers that other crops may be more profitable than tobacco.

## INTRODUCTION

Tobacco is a killer plant and the greatest single current public health threat. The annual global burden of disease reflected in premature death and disability attributable to tobacco use is more than 157 million disability-adjusted life years.^1^ These disability-adjusted life years represent approximately 4% of all premature deaths and disability of all diseases combined.^2^ Smoking causes more than 7 million deaths annually, of which 90% are the result of direct tobacco use and approximately 10% a result of passive smoking.^2,3^ More than 1.1 billion people smoke worldwide, with 80% of smokers living in low- and middle-income countries where the prevalence of smoking is increasing compared with that of high-income countries.^3^ Reducing tobacco use can be based on strategies that diminish demand (ie, consumption) and/or supply (ie, availability). Most efforts at tobacco control concentrate on limiting demand with the primary intent of modifying the mindset and behavior of smokers or potential smokers through such actions as high taxation of tobacco products, bans on cigarette promotions, requirements for warning labels, smoke-free public spaces and workplaces, public education, and programs to support quitting.^4-8^ In tobacco control, supply-side measures that are aimed at reducing availability, such as tobacco prohibition, bans on commercial tobacco trade, and replacing the growing of tobacco with other crops (crop diversification or substitution),^9-15^ have received relatively less attention. This is based on the premise that supply-side interventions would be less effective in reducing the consumption of tobacco compared with demand-side interventions.^16^ However, a more realistic reason for less enthusiasm with supply side–based interventions is that most, if not all, supply control requires the use of state power to intervene in the tobacco market so as to protect the public health by either using the “carrot” (eg, through the use of subsidies and buyouts)^17-21^ or the “stick” (eg, through legislation and enforcement).^9-13,22^

**CONTEXT**

**Key Objective** To analyze the factors that influence whether farmers are willing to stop growing tobacco and opt for other crops.
**Knowledge Generated** The factors influencing the willingness to stop growing tobacco were health related as well as economic. Tobacco farmers who agreed that tobacco is harmful to health (eg, that it causes respiratory disease) were willing to stop growing the crop, whereas tobacco farmers whose annual income from tobacco was more than $300 USD and those who said that tobacco is more profitable compared with other cash crops were not willing to stop growing tobacco.
**Relevance** To increase the willingness of farmers to stop growing tobacco, there is a need to increase the knowledge of farmers regarding the harmful effects of tobacco and to promote alternative crops that are more profitable than tobacco. Furthermore, research is needed on the costs and benefits of tobacco compared with alternative crops.

Nevertheless some authorities argue that control of the supply side is as important as that of the demand side if we are to sustain or even eliminate the use of tobacco.^23-26^ Efforts to implement comprehensive tobacco control policies culminated in the adoption of the WHO Framework Convention on Tobacco Control (FCTC) in 2003.^7^ The FCTC suggested that a critical element of supply control is the provision of technical and financial assistance to ease the transition for tobacco growers to economically viable alternatives.^27^ In this work, we attempt to explain the views of tobacco farmers on crop diversification, which means replacing the growing of tobacco with crops that are less or nondetrimental to public health. The understanding of such views is useful in informing policy and practice about measures aimed at replacing the growing of tobacco with other crops.^28^

## METHODS

The study was conducted in Kanungu district of southwestern Uganda. Commercial tobacco growing in Uganda began in 1927 and the crop is currently grown in 25 of 112 districts. Approximately 75,000 farmers grow tobacco in Uganda and the crop has a market value of more than $80 million USD in 2013,^29^ making tobacco one of Uganda’s top 10 revenue sources. There are three commercially grown tobacco types, including flue-cured Virginia, burley (air-cured), and dark fire–cured tobacco. Tobacco is one of the most regulated crops in the country. Areas for production are regulated, as are the inputs to be used or prohibited, leaf buying, and tobacco types. Small-scale farmers who are registered contract with one of the five tobacco companies in Uganda to provide seedlings, inputs, and training for their contracted farmers. Uganda ratified the WHO FCTC in 2007 and passed the tobacco control act in 2015.^30^ Kanungu district has a total land area of 1,228 km^2^ with a population of 252,144 people, according to the 2014 population census. Agriculture is the mainstay of the district’s economy, as is the case for a majority of Ugandan districts. Crops grown in the district include tobacco, coffee, rice, beans, cassava, vegetables, and sweet potatoes. In addition, animals such as cows, goats, chickens, and pigs are reared. Tobacco grown in the district is primarily sold to a few tobacco companies that train and facilitate farmers that grow the crop. Registered small-scale farmers contract with the tobacco companies to buy the leaf, and tobacco companies, in turn, provide seedlings, fertilizers, agricultural loans, and training for their contracted farmers. Most households with large tobacco farms grow other crops, such as coffee, rice, and vanilla, with support provided by the National Agricultural Advisory Services. However, the support to farmers provided by the National Agricultural Advisory Services is irregular, inferior, and meager compared with the support and incentives from the tobacco industry, and as a result little effort is given to growing other crops as they are considered supplementary to tobacco production.

### Sampling and Data Collection

Twelve villages were randomly selected from a listing of 508 villages in the district. From each village, 44 households were selected randomly from a list of all registered tobacco farmers. Both steps used computer-generated random numbers. Between April and May 2013, trained research assistants orally interviewed the head of these household. Research was approved by the Makerere University School of Public Health institutional review board. Informed consent was obtained before interviews took place.

Data were collected on sociodemographic characteristics, such as age, sex, total land acreage, land used for growing tobacco and other crops as of last season, income from tobacco, income from other crops, alternative sources of income, number of years growing tobacco, religion, occupation, and level education. Data were also collected on the use of tobacco, beliefs about the health effects of tobacco (on cancer and respiratory and heart disease as well as the effect on pregnancy and effects of passive smoking), and beliefs about the economic benefits of tobacco (whether tobacco was easy to grow, easy to sell, and profitable compared with other crops). Heads of households were also asked whether they were willing to stop growing tobacco and to replace it with other crops.

### Analysis

We used bivariable analysis with χ^2^ or Fisher’s exact test by two-tailed tests to compare proportions. Crude prevalence ratios and the 95% CIs were calculated after bivariable analysis. To identify independent predictors of willingness to stop growing tobacco, we used the log-binomial model with a clustered robust variance. All variables that were significant via bivariable analysis (*P* < .05) were used in the multivariable analyses. To control for multicollinearity in the data, we constructed a correlation matrix between variables, and for extreme correlations (> 0.55) between two variables, one was dropped from the regression. The variable left in the model was that which was more logically likely to influence the willingness to continue or stop growing tobacco. We calculated adjusted prevalence ratios after multivariable analysis. We used the statistical software package STATA 12 (STATA, College Station, TX; Computing Resource Center, Santa Monica, CA) in the analysis, taking into consideration the cluster effect at the village level.

## RESULTS

Of the sample, 53 participants (10.0%) were female and 296 (56.1%) were older than age 40 years. Slightly more than 90% was Christian and 323 respondents (61.2%) had received a primary education. Most heads of household were full-time farmers (445; 84.3%) with no supplementary employment.

Respondents were predominantly small-scale farmers, with approximately one half (51.7%) growing tobacco on less than 1 acre of land. Only 202 farmers (38.3%) grew coffee or rice to supplement their income from tobacco. In addition to crops for sale, almost every family grew crops for consumption (eg, beans, sweet potatoes, and cassava). Income from tobacco sales, as of the previous season, was meager, with more than 68% earning less than 1,500,000 Uganda shillings (about $440 USD). Overall, 86 participants (16.3%) were consumers of tobacco via smoking cigarettes (n = 62), chewing tobacco (n = 6),^6^ or pipe smoking (n = 18).^18^

### Willingness to Stop Growing Tobacco

Of the 528 tobacco growers interviewed, 198 (37.5%) answered in the affirmative when asked if they would be willing to grow another cash crop in place of tobacco. Ninety-two respondents (17.4%) mentioned coffee as a replacement for tobacco and 106 (53.5%) cited rice. Three hundred twenty-nine participants (62.3%) said they are not willing to replace tobacco with another cash crop. Reasons offered for this unwillingness were that tobacco is highly profitable (172; 32.6%), the soil cannot sustain any crop other than tobacco (175; 33.1%), and the weather in Kanungu is unpredictable for other crops (22; 4.2%). Drawing from the study population, when asked whether tobacco was easy to grow, sell, and highly profitable compared with other crops, 301 respondents (57%) said it was easy to grow, 348 (65.9%) said it was easy to sell, and 441 (83.5%) said that tobacco was highly profitable.

### Determinants of Willingness to Stop Growing Tobacco

Of sociodemographic variables, willingness to stop growing tobacco was more likely if the household has been growing tobacco for less than 5 years, tobacco was grown on less than 1 acre of land, the household was already growing other cash crops in addition to tobacco, and the household received less than $300 USD from tobacco sales and had additional income from crops other than tobacco or other noncrop sources (Table 1). Age of the head of household, education, religion, occupation, and marital status did not influence the willingness to stop growing tobacco.

**TABLE 1:**
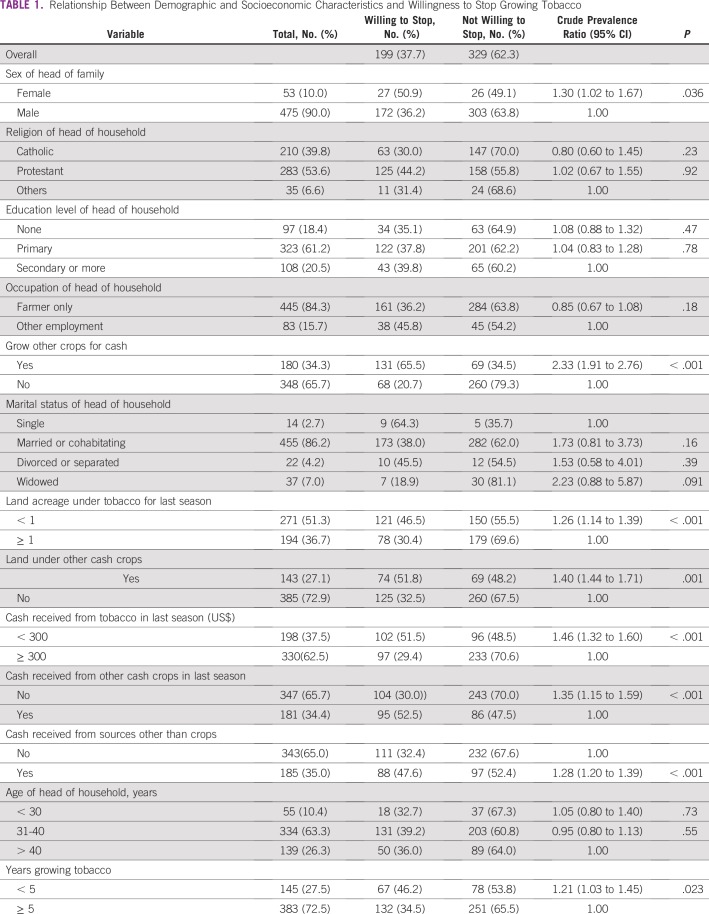
Relationship Between Demographic and Socioeconomic Characteristics and Willingness to Stop Growing Tobacco

Associations between the use of tobacco, health-related effects, and willingness to stop growing tobacco are shown in Table 2. Willingness to stop growing tobacco was more likely if the respondent said that the use of tobacco is harmful to pregnancy or causes heart disease, respiratory disease, or cancer. Current use of tobacco from smoking cigarettes or a pipe or through chewing did not influence the willingness to stop growing tobacco.

**TABLE 2:**
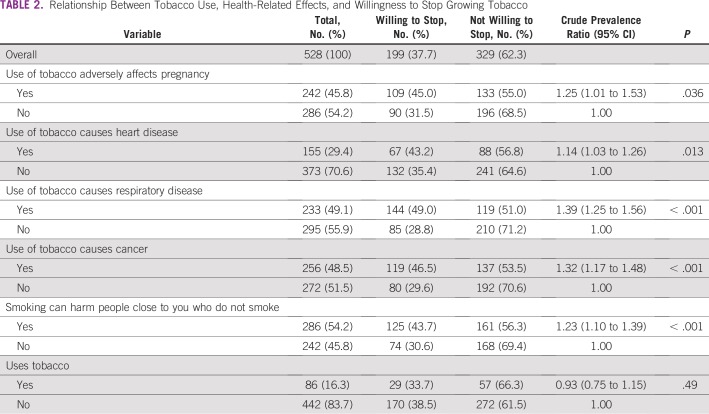
Relationship Between Tobacco Use, Health-Related Effects, and Willingness to Stop Growing Tobacco

Attitudinal beliefs influencing the willingness to stop growing tobacco are listed in Table 3. Agreeing that tobacco was easy to grow, sell, or that it was profitable reduced the likelihood of willingness to stop growing the crop. Similarly, agreeing that soils in Kihihi cannot support the growth of other cash crops decreased the likelihood of willingness to stop growing the crop.

**TABLE 3:**
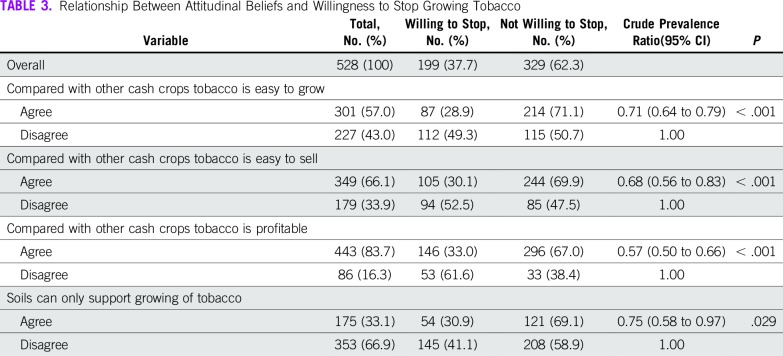
Relationship Between Attitudinal Beliefs and Willingness to Stop Growing Tobacco

### Independent Predictors of Willingness to Stop Growing Tobacco

Independent predictors of the willingness to stop growing tobacco (Table 4) were as follows: saying that tobacco use causes respiratory disease and receiving less than $300 USD from tobacco sales in the previous season. Agreeing that tobacco is profitable decreased the likelihood of willingness to stop growing tobacco.

**TABLE 4:**
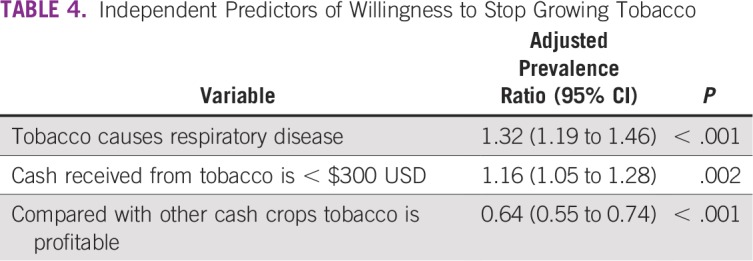
Independent Predictors of Willingness to Stop Growing Tobacco

## DISCUSSION

The current study has demonstrated that fewer than one in three tobacco farmers are willing to stop growing tobacco and switch to other crops. Reasons for the lack of support for diversification were both economic and health related. The greater the amount of money earned from tobacco, the less the likelihood of diversification. Respondents who reported that tobacco is profitable compared with other crops were not willing to diversify. Knowing that tobacco use is associated negative health consequences, such respiratory diseases, was an independent predictor of the willingness to stop growing of tobacco.

The implications of these findings are several if agricultural diversification is to be used as a successful tool for tobacco control. First, the results imply that measures aimed at increasing the knowledge of farmers regarding the harmful effects of tobacco will increase the likelihood that tobacco farmers will consider diversification. This is important as only one in two of farmers associate tobacco use with negative health consequences. Second, because the perceived and/or real financial benefits attributable to tobacco decrease the likelihood that tobacco farmers will diversify, there is a need to promote alternative crops that are more profitable, easier to grow, and easier to sell compared with tobacco. Clearly, getting viable alternative crops to replace tobacco is not straightforward. Additional research is needed on the costs and benefits of tobacco compared with alternative crops to particularly demonstrate that income from crop substitution can exceed that of growing tobacco.^31,32^

Furthermore, because tobacco is regarded as a killer crop as well as a demerit good that harms the consumer through active use and has negative externalities of harming society through passive smoking, direct government intervention is justifiable to tip the financial and economic balance in favor of alternative crops. Such measures may include subsidizing the growth of other crops,^18,21^ price support for other crops, and/or giving cash handouts (buyouts) to tobacco farmers so that they stop growing the crop.^17,19,20^ Moreover, government intervention may take the form of a guaranteed market for alternative crops and ensuring the provision of seeds, farm equipment, and fertilizers for alternative crops as is currently done for tobacco.^18,21^

The current study is limited mainly as a result of its cross-sectional nature. This means that it is difficult to establish cause and effect. For example, price fluctuations of other crops compared with tobacco could lead the respondents to overestimate the economic contribution of tobacco.

Nevertheless, this study provides useful information about the determinants of crop diversification, and the results are similar to those reported in different diverse settings.^14-20,28-32^ These data and the implications are therefore deemed generalizable beyond the study settings.

## References

[1] GBD 2015 Risk Factors Collaborators Global, regional, and national comparative risk assessment of 79 behavioural, environmental and occupational, and metabolic risks or clusters of risks, 1990-2015: A systematic analysis for the Global Burden of Disease Study 2015 Lancet 388 1659 1724 2016 Erratum: Lancet 389:e1, 2017 27733284 10.1016/S0140-6736(16)31679-8 PMC5388856

[2] GBD 2015 Tobacco Collaborators Smoking prevalence and attributable disease burden in 195 countries and territories, 1990–2015: A systematic analysis from the Global Burden of Disease Study 2015 Lancet 389 1885 1906 2017 Erratum: Lancet 390:1644, 2017 28390697 10.1016/S0140-6736(17)30819-X PMC5439023

[3] World Health Organization Fact sheets: Tobacco http:// www.who.int/mediacentre/factsheets/fs339/en/

[4] World Health Organization WHO report on the global tobacco epidemic 2015 https://www.who.int/tobacco/global_report/2015/en/

[5] Jha P Peto R Global effects of smoking, of quitting, and of taxing tobacco N Engl J Med 370 60 68 2014 24382066 10.1056/NEJMra1308383

[6] The World Bank Curbing the Epidemic: Governments and the Economics of Tobacco Control Washington, DC World Bank 1999

[7] World Health Organization Building Blocks for Tobacco Control: A Handbook Geneva, Switzerland World Health Organization 2004

[8] Wang MP Wang X Lam TH et al The tobacco endgame in Hong Kong: Public support for a total ban on tobacco sales Tob Control 24 162 167 2015 24046209 10.1136/tobaccocontrol-2013-051092

[9] Proctor RN Why ban the sale of cigarettes? The case for abolition Tob Control 22 i27 i30 2013 suppl 1 23591501 10.1136/tobaccocontrol-2012-050811 PMC3632991

[10] Daynard RA Doing the unthinkable (and saving millions of lives) Tob Control 18 2 3 2009 19168478 10.1136/tc.2008.028308

[11] Chapman S The case for a smoker’s license PLoS Med 9 e1001342 2012 23152726 10.1371/journal.pmed.1001342 PMC3496663

[12] Shahab L West R Public support in England for a total ban on the sale of tobacco products Tob Control 19 143 147 2010 20378589 10.1136/tc.2009.033415

[13] Khoo D Chiam Y Ng P et al Phasing-out tobacco: Proposal to deny access to tobacco for those born from 2000 Tob Control 19 355 360 2010 20876075 10.1136/tc.2009.031153 PMC2978941

[14] Panchamukhi PR Agricultural diversification as a tool of tobacco control https://www.who.int/tobacco/media/en/PANCHIMUKHI2000X.pdf

[15] Jha P Chaloupka F The supply-side effects of tobacco control policies Tobacco Control Policies in Developing Countries New York, NY Oxford University Press 2000

[16] Wales M Searching for alternative crops to replace or enhance tobacco in Canada “The search for the holy grail” http://nuffield.ca/wp-content/uploads/2009/09/MarkWalesReport.pdf

[17] Chibwana C Fischer M Cropland allocation effects of agricultural input subsidies in Malawi World Dev 40 124 133 2012

[18] Dohlman E Foreman L Da Pra M The post-buyout experience: Peanut and tobacco sectors adapt to policy reform http://www.ers.usda.gov/Publications/EIB60/EIB60.pdf

[19] Brown B The end of the tobacco transition payment program in North Carolina https://tobacco.ces.ncsu.edu/wp-content/uploads/2013/11/The-End-of-the-Tobacco-Transition-Payment-Program.pdf?fwd=no

[20] Khumalo CCM Can farmers diversify from growing tobacco in Zimbabwe? https://stud.epsilon.slu.se/6277/1/khumalo_c_131114.PDF

[21] Callard C Thompson D Collishaw N Transforming the tobacco market: Why the supply of cigarettes should be transferred from for-profit corporations to non-profit enterprises with a public health mandate Tob Control 14 278 283 2005 16046692 10.1136/tc.2005.011353 PMC1748051

[22] Young D Borland R Changing the tobacco use management system: Blending systems thinking with actor–network theory Rev Policy Res 29 251 279 2012

[23] Callard CD Collishaw NE Supply-side options for an endgame for the tobacco industry Tob Control 22 Suppl 1 i10 i13 2013 23591497 10.1136/tobaccocontrol-2012-050863 PMC3632987

[24] Kessler D A Question of Intent: A Great American Battle With a Deadly Industry New York, NY Public Affairs 2001

[25] Thomson G Wilson N Blakely T et al Ending appreciable tobacco use in a nation: Using a sinking lid on supply Tob Control 19 431 435 2010 20876079 10.1136/tc.2010.036681

[26] US Department of Health and Human Services The Health Consequences of Smoking: 50 Years of Progress: A Report of the Surgeon General Atlanta, GA US Department of Health and Human Services 2014

[27] World Health Organization Parties to the WHO Framework Convention on Tobacco Control http://www.who.int/fctc/ signatories_parties/en/

[28] Altman DG Zaccaro DJ Levine DW et al Predictors of crop diversification: A survey of tobacco farmers in North Carolina (USA) Tob Control 7 376 382 1998 10093171 10.1136/tc.7.4.376 PMC1751454

[29] Ministry of Health Uganda Global adult tobacco survey: Country report 2013 health.go.ug/content/gats-uganda-country-report-final

[30] Republic Of Uganda The tobacco control ACT 2015 health.go.ug/download/file/fid/1110

[31] Li VC Wang Q Xia N et al Tobacco crop substitution: Pilot effort in China Am J Public Health 102 1660 1663 2012 22813422 10.2105/AJPH.2012.300733 PMC3482040

[32] Keyser JC Crop substitution and alternative crops for tobacco www.who.int/tobacco/framework/cop/events/2007/keyser_study.pdf

